# Voltammetric Immunosensor to Track a Major Peanut Allergen (Ara h 1) in Food Products Employing Quantum Dot Labels

**DOI:** 10.3390/bios11110426

**Published:** 2021-10-29

**Authors:** Maria Freitas, Henri P. A. Nouws, Cristina Delerue-Matos

**Affiliations:** REQUIMTE/LAQV, Instituto Superior de Engenharia do Porto, Instituto Politécnico do Porto, Rua Dr. António Bernardino de Almeida 431, 4200-072 Porto, Portugal; maria.freitas@graq.isep.ipp.pt (M.F.); cmm@isep.ipp.pt (C.D.-M.)

**Keywords:** Ara h 1, biosensor, electrochemical immunosensor, food allergy, nanolabel, peanut allergy, SPCE, quantum dots

## Abstract

Tracking unreported allergens in commercial foods can avoid acute allergic reactions. A 2-step electrochemical immunosensor was developed for the analysis of the peanut allergen Ara h 1 in a 1-h assay (<15 min hands-on time). Bare screen-printed carbon electrodes (SPCE) were used as transducers and monoclonal capture and detection antibodies were applied in a sandwich-type immunoassay. The short assay time was achieved by previously combining the target analyte and the detection antibody. Core/shell CdSe@ZnS Quantum Dots were used as electroactive label for the detection of the immunological interaction by differential pulse anodic stripping voltammetry. A linear range between 25 and 1000 ng·mL^−1^ (LOD = 3.5 ng·mL^−1^), an adequate precision of the method (V_x0_ ≈ 6%), and a sensitivity of 23.0 nA·mL·ng^−1^·cm^−2^ were achieved. The immunosensor was able to detect Ara h 1 in a spiked allergen-free product down to 0.05% (m/m) of peanut. Commercial organic farming cookies and cereal and protein bars were tested to track and quantify Ara h 1. The results were validated by comparison with an ELISA kit.

## 1. Introduction

Awareness about food allergies has risen globally because of the increase of the number of reported allergic occurrences [1]. The ingestion of allergic substances that are not mentioned on product labels can potentially be harmful to sensitized individuals [2]. The related symptoms are generally due to the action of immunoglobulin E (IgE), varying from mild to severe systemic reactions, namely cutaneous, digestive and/or cardiovascular complications, and respiratory difficulties with anaphylactic shock that require emergency treatments [2,3]. Diagnosis can be complex as the symptoms and reactions vary from person to person and depend on the food exposure and/or the quantity of ingested allergen [4]. Legumes, such as peanuts, are included in the class of allergens of high relevance in food allergies [5] and can cause severe allergic attacks since ingestion of trace amounts can be life-threatening. Presently, the appropriate patients’ treatment is the avoidance of peanut-containing products.

The plant-based seed storage protein *Arachis hypogaea* (Ara h) is an important predictor of clinical reactivity to peanut allergic reactions [6]. Since the incidence of peanut allergy is increasing, tracking Ara h 1, a major peanut allergen (cupin; vicilin-type 7S globulin), in food products can prevent acute allergic reactions [7]. Subjecting commercial food to an effective quality control is essential to identify non-compliance products, related to fraud or food adulteration, and can avoid undesirable health disorders [8]. As required by legislation, commercial food product labels must declare the presence of peanuts, even when this resulted from accidental exposure or uncontrolled cross-contamination in the production facilities [9,10]. Effective methodologies that can increase the testing frequency and tighten food control to ensure improved product quality for the consumer are therefore of utmost importance. Biomolecular-based assays (especially the ones that use aptamers, DNA and/or proteins) have a remarkable impact on the control of allergens in commercial food products [11,12]. It has been proven that point-of-need biosensing devices are feasible platforms for rapid allergen detection [13,14]. Electrochemical biosensors can be highlighted as representative examples of these devices [15,16,17]. The analysis of food allergens, adulterants and functional foods by cutting-edge advances at different molecular levels provides new insights in biosensing strategies [18,19]. Furthermore, innovative and user-friendly immunochemical tools have been developed to accomplish food control and identify fraudulent food manufacturing technologies [20,21].

The use of nanotechnological advances in the biosensing field is part of the state-of-the-art research frontiers in immunosensing [22]. Quantum dots (QDs) are nanometer-scale semiconductor crystals and are promising labels that can be applied for signal amplification in electrochemical immunosensors. Because of their inherent electroactivity, their use results in a considerably enhanced analytical performance, especially when combined with the high sensitivity of anodic stripping voltammetry (ASV). Thus, rapid and cost-effective sensors using QD labels can significantly improve the analysis strategy. In this work we used cadmium-containing QDs. These QDs are dissolved, and the released cadmium ions are analysed using ASV; first the ions are reduced to metallic cadmium by applying a negative potential and then the cadmium is stripped back into the solution through an anodic potential scan. In traditional ASV, which dates for almost a century now, mercury was used for the analysis of heavy metals after the formation of an amalgam, but because its toxicity it is highly recommended not to be used, neither in its elemental form nor in the form of salts [23]. Therefore, several other materials have been studied when carbon electrode surfaces are used. The released cadmium ions can be directly measured on an SPCE, but a rather low sensitivity is obtained [24]. Bismuth was found to provide the highest sensitivity when compared with bare SPCE and antimony and/or bismuth/antimony films. Bismuth forms fused alloys with heavy metals, which are analogous to amalgams [25]. Between the various ways to modify the electrode surface with bismuth films, in situ plating simplifies and shortens the experimental procedure (as no separate bismuth-plating step is required) and is well suited [25,26,27]. In a distinct approach, graphite electrode covered with Sb/Sn nanoparticles formed in-situ by reduction of the embedded precursors (Sb_2_O_5_/SnO_2_) was also applied. Although a higher sensitivity is reported compared to bismuth, antimony, and tin electroplating film electrodes, additional platform preparation and precursor reagents are required, leading to an expensive and time-consuming method [28]. There are only a few works regarding the development of electrochemical immunosensors applying QDs as detection labels to ensure food safety: a 3D printed microcell to evaluate adulterated ewe/goat’s cheese species with cow’s milk [28], a lab-on-a-membrane device to detect milk adulteration [29], direct culture-free analysis of *Salmonella* in milk [30], biointerface platforms for the food toxin aflatoxin B_1_ [31,32] and determination of the mycotoxin fumonosin in corn [33]. In this context, QDs constitute an alternative to the traditional enzymatic probes, avoiding their major drawbacks (e.g., thermal instability, substrate addition, cross-reactivity with sample components) [34,35]. Besides this, the simplified assay’s workflow when QDs are used and the higher sensitivity of the analysis, could make this strategy surpass standardized immunoassays, molecular biological or chromatographic methods [36].

To enforce the compliance of food labelling legislation, this work presents a sandwich-type electrochemical immunosensor (assay time: 1 h) for the determination of Ara h 1 in commercial foods. The assay was carried out on (bare) SPCEs (biomodified with monoclonal anti-Ara h 1 capture antibodies). Biotinylated detection antibodies (previously mixed with the target analyte) and streptavidin-coated core/shell CdSe@ZnS QDs were used to perform the two-step immunoassay. Differential pulse anodic stripping voltammetry (DPASV) was employed to detect the immunological interaction by the dissolution, preconcentration and stripping of the cadmium contained in the QDs. The analytical signal was obtained after the addition of HCl (acidic dissolution of the QDs to release cadmium ions) and an acetate buffer containing Bi(III) (to obtain the bismuth film on the SPCE’s surface). A preconcentration step (electrochemical deposition) was applied in which the dissolved (ionic) metal label was reduced to its solid state. Then an anodic (stripping) potential scan was applied to determine cadmium at a peak potential (*E*_p_) of about −0.9 V. The resulting peak current intensity (*i*_p_) increases with increasing target analyte concentration and was quantitatively related with the presence of Ara h 1.

## 2. Materials and Methods

### 2.1. Apparatus and Electrodes

A Metrohm Autolab potentiostat/galvanostat (PGSTAT204) controlled by NOVA software (v.1.11) was used for the electrochemical measurements (Metrohm DropSens, Oviedo, Spain). Screen-printed carbon electrodes (4-mm carbon working electrode (electroactive area: 0.079 cm^2^), silver pseudoreference electrode and carbon counter electrode (SPCE, DRP-110)) were connected to a DRP-CAC interface (Metrohm DropSens). A HulaMixer^TM^ Sample Mixer (Invitrogen–Thermo Fisher Scientific, Oslo, Norway) and a Fresco^TM^ microcentrifuge (Heraeus-Fresco 21, Thermo Fisher Scientific, Osterode am Harz, Germany) were used for sample preparation. An ELISA kit was used to assess the accuracy of the assay’s results and the analysis was performed using a multi-mode microplate reader (Synergy HT W/TRF, BioTek Instruments, Winooski, VT, USA). Data were treated with Gen5 Version 2.0 data analysis software (BioTek Instruments). A Quanta 400FEG ESEM/EDAX Genesis X4 M system (FEI, Hillsboro, OR, USA) located at the Centro de Materiais da Universidade do Porto (CEMUP) in Porto, Portugal was used to obtain the SEM images.

### 2.2. Reagents and Solutions

Mouse IgG_1_ monoclonal anti-Ara h 1 antibody (capture antibody (CAb), clone 2C12), biotinylated monoclonal anti-Ara h 1 IgG_1_ antibody (detection antibody (DAb), clone 2F7) and naturally purified Ara h 1 were acquired from Indoor Biotechnologies. These reagents were also used to perform the ELISA assay for comparison purposes. The Qdot^TM^ 655 streptavidin conjugate (QD) was obtained from Invitrogen–Thermo Fisher Scientific. Acetic acid, bismuth(III) ICP standard and sodium hydroxide were purchased from Merck (Darmstadt, Germany). Bovine serum albumin fraction V (BSA), tris(hydroxymethyl)aminoethane (Tris) and hydrochloric acid were purchased from Sigma-Aldrich (St. Louis, MO, USA).

The working solutions (BSA, antibodies, antigen and QDs) were prepared daily in a 0.1-M Tris-HCl buffer (pH 7.4, Tris buffer). A washing buffer (Tris-0.01% Tween 20, pH 7.4 (Tris-T)) was used between the incubation steps and an extraction buffer (0.1 M Tris-HCl, pH 8.5) was used in the sample preparation. A Bi(III) solution (1.0 mg·L^−1^) was prepared in a 0.1-M acetate buffer (pH 4.5). Non-target proteins and other food ingredients were used to evaluate the selectivity of the sensor and to assess their possible interference in the analysis. All solutions were prepared in ultrapure water (resistivity = 18.2 MΩ·cm).

### 2.3. Immunosensor/Assay Development

**Figure 1:**
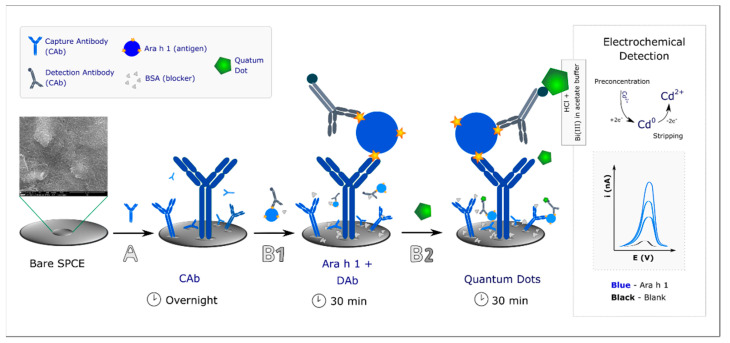
Schematic representation of the construction of the electrochemical immunosensor. (**A**) Incubation with CAb, followed by incubation with (**B1**) a mixture solution containing the allergen (Ara h 1) and DAb, and (**B2**) a Quantum Dot solution. For electrochemical detection, after addition of HCl, to dissolve the QD, and Bi(III), to form a bismuth film, differential pulse anodic stripping voltammetry was used. Representative voltammograms of increasing Ara h 1 concentrations are exemplified.

Figure 1 illustrates the transducer modification and the immunoassay. Bare SPCEs were biomodified by placing (A) a 5-µL aliquot of a capture antibody solution (CAb, 10 µg·mL^−1^) on the working electrode (WE) surface (incubated overnight, at 4 °C in a moist atmosphere. Like this the CAb were physisorbed on the WE. Then, the SPCE was washed using Tris-T to remove the weakly absorbed or unbound antibodies.

The optimized immunoassay protocol (B) consisted of two steps:

(B1) a 40-µL aliquot of a previously prepared mixture (containing Ara h 1 (target molecule or sample) and DAb (250× dilution in Tris BSA 1.0% (m/V)) was incubated on the SPCE (30 min);

(B2) After washing with Tris-T to remove the excess or unbound biomolecules, a 40-µL aliquot of a QD solution (5.0 nM in Tris BSA 2.0% (m/V) was placed on the transducer surface (30 min). The SPCE was then washed with ultrapure water and totally dried using a nitrogen flow before connecting the electrode to the equipment for DPASV measurements.

### 2.4. Electrochemical Detection

For the electrochemical analysis, a sequential addition of 5 µL of HCl (1 M) and 40 µL of the Bi(III) solution (1.0 mg·L^−1^, prepared in a 0.1-M acetate buffer (pH 4.5)) were placed on the sensor surface. HCl was used to release cadmium ions from the QDs and also to prevent the precipitation of bismuth. DPASV was performed by applying a constant potential of +1.00 V for 60 s (to activate the WE and increase the electroactive area), followed by a potential of −1.10 V for 300 s (to reduce and preconcentrate cadmium ions and to obtain a bismuth film, also formed simultaneously during this step) for the sensitive detection of cadmium. The potential was then swept from −1.00 V to −0.70 V to strip the cadmium into the solution (*E*_p_ ≈ −0.9 V). The DPV parameters were: pulse amplitude −0.05 V; step potential −0.01 V; modulation time—0.01 s; interval time—0.1 s [25,26].

### 2.5. Food Sample Preparation

A set of commercial food products was obtained from local supermarkets. The optimized immunosensor was used to analyse legumes (soybean, lupin), other allergenic foods (almond, sesame, wheat flour, hazelnut), allergen-free products (cookies, energetic cereal bar) and peanut-containing foods (cookies and cereal and protein bars).

Samples were ground and homogenized (3×, 20 s) and 1.0 g of each powdered sample was mixed with 10.0 mL of extraction buffer. The suspensions were then stirred continuously for 60 min at 25 °C [37]. The resulting suspensions were centrifuged for 5 min at 5000 rpm (4 °C) and a 1-mL aliquot was taken and further centrifuged for an additional 10 min at 10,000 rpm [38]. The final extracts were diluted (1000×) in Tris-BSA 1.0% (m/V). A 40-μL aliquot of the resulting solution was used to perform the electrochemical immunoassay.

## 3. Results and Discussion

### 3.1. Method Development and Optimization

To achieve selective and sensitive analysis of Ara h 1 in commercial foods, a detailed and extensive optimization study was carried out. The CAb was immobilized by physical adsorption on the WE in accordance with our previous study [26]. This immobilization method was used because it allows an adequate performance without needing additional procedures and reagents, such as the ones involved in covalent binding [39] or self-assembled monolayers [40], leading to simpler and cheaper immunosensor construction. Additionally, a highly specific IgG_1_ monoclonal anti-Ara h 1 antibody was employed to efficiently recognize and immunocapture the specific Ara h 1 epitopes, in combination with a biotinylated monoclonal anti-Ara h 1 IgG_1_ antibody (both capable of recognizing non-overlapping specific binding sites of the target protein) in a sandwich-type format assay. The proposed immunoassay consisted of incubation of the target analyte and the biotinylated DAb that was subsequently linked to streptavidin-QDs through the biotin-streptavidin affinity process. The electrochemical detection was performed using a fully optimized protocol [25,26]: screen-printed electrodes as transducers and Strep-QD as electroactive label.

The following experimental parameters involved in the preparation of the immunosensor were optimized/studied: CAb, DAb and QDs concentrations, assay format and time, and non-specific interactions. To select the optimum value, the signal-to-blank (S/B) ratio was considered, in the presence (S, signal; 250 ng·mL^−1^ Ara h 1) and absence (B, blank; 0 ng·mL^−1^ Ara h 1) of the target analyte.

**Figure 2:**
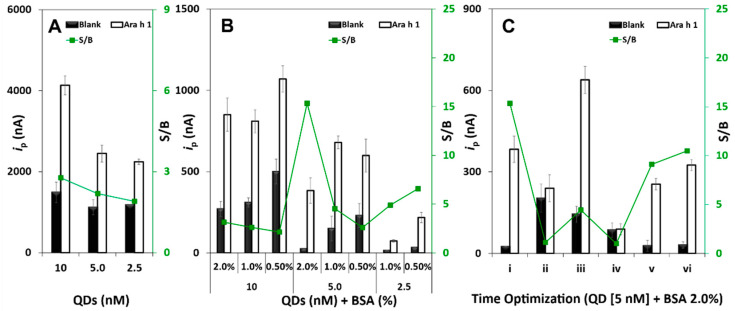
Results of the study/optimization in the absence (“B”, 0 ng·mL^−1^) and in the presence (S, 250 ng·mL^−1^) of Ara h 1 and the corresponding S/B ratio value for the following experimental parameters: (**A**) QDs concentration. (**B**) Addition of BSA to the tested QD solutions. (**C**) Assay format and time optimization using distinct assay strategies: **i**—step-by-step; **ii**—pre-incubation of DAb + QDs; **iii**—pre-incubation of target + DAb; **iv**—pre-incubation of target + DAb + QDs; **v**—pre-incubation of target + DAb; **vi**—(No blocker) pre-incubation of target + DAb (Experimental conditions: blocker (Tris BSA 2.0%), DAb (250× dilution in Tris BSA 1.0% (m/V), QDs (5.0 nM in Tris BSA 2.0% (m/V)).

To optimize the detection of Ara h 1, different QD concentrations were tested (10, 5.0, and 2.5 nM). As expected, the highest analytical signal (i.e., peak current intensity (*i*_p_)) was obtained for the 10 nM solution. Besides this, relatively high blank signals were obtained for all the tested concentrations (Figure 2A), which was probably due to non-specific adsorptions. To minimize this effect and to allow a more efficient target detection, different concentrations of BSA (2.0, 1.0 and 0.50 % (m/V)) were added to the QD solutions. As can be observed in Figure 2B, significant differences were observed when compared to the previous study. Considering the signal-to-blank (S/B) ratio, a 5.0-nM QD + BSA 2.0% (m/V) solution demonstrated the most satisfactory performance of the sensor and was selected for the subsequent work.

To obtain faster results, distinct assay strategies based on a combination of steps were tested (Table S1): **i**—step-by-step: (1) blocker, 30 min; (2) target, 60 min; (3) DAb, 60 min; (4) QDs, 30 min; **ii**—(1) blocker, 30 min; (2) target, 60 min; (3) pre-incubation of DAb + QDs, 60 min; **iii**—(1) blocker, 30 min; (2) pre-incubation of target + DAb, 60 min; (3) QDs, 30 min; **iv**—(1) blocker, 30 min; (2) pre-incubation of target + DAb + QDs, 60 min; **v**—(1) blocker, 30 min; (2) pre-incubation of target + DAb, 30 min; (3) QDs, 30 min; **vi**—(No blocker) (1) pre-incubation of target + DAb, 30 min; (2) QDs, 30 min. (Experimental conditions: blocker (Tris BSA 2.0%), target (Ara h 1: 0 and 250 ng·mL^−1^), DAb (250× dilution in Tris BSA 1.0% (m/V), QDs (5.0 nM in Tris BSA 2.0% (m/V)).

As can be observed in Figure 2C, strategies **i** and **vi** provided the highest S/B ratios. Because strategy **i** requires a much longer assay time (3 h) and is less user-friendly compared to strategy **vi** (1 h), the latter option was chosen to proceed with the development of the assay. The remarkable performance of this 2-step assay can be justified by the presence of BSA in the mixture (Ara h 1 + DAb) and QD solutions, effectively blocking residual binding sites on the transducer surface, thus not requiring an initial blocking step typically employed in immunoassays.

**Figure 3:**
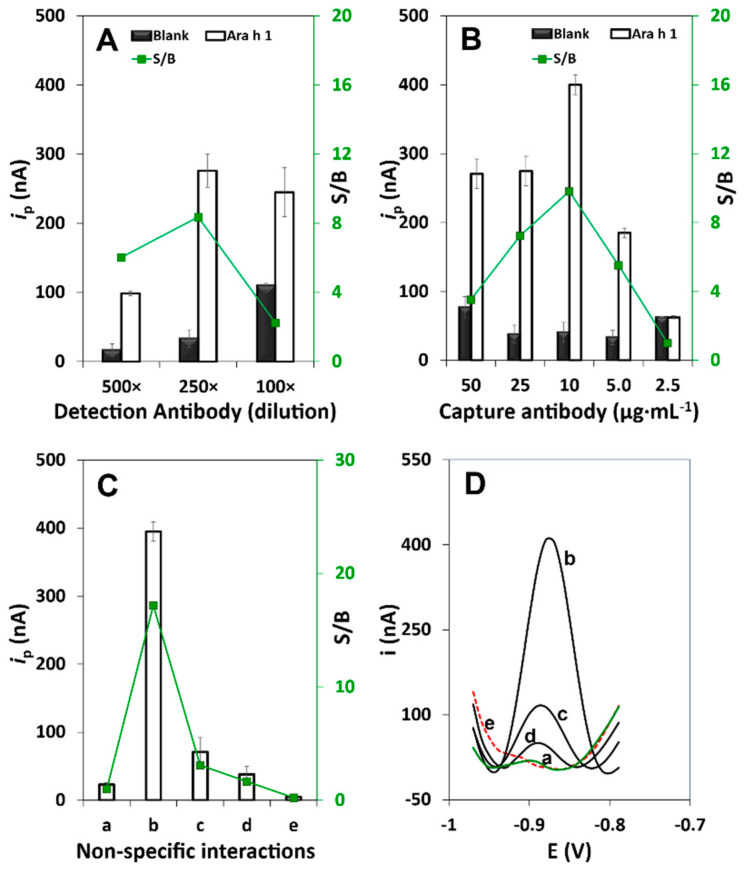
Obtained *i*_p_ values by DPASV in the absence (“B”, 0 ng·mL^−1^) and in the presence (S, 250 ng·mL^−1^) of Ara h 1 and the corresponding S/B ratio value for the optimization of (**A**) DAb dilution factor and (**B**) CAb concentration. (**C**) Study/evaluation of non-specific interactions and biosensor performance for the optimized and complete immunoassay: *i*_p_ values obtained in the (**a**) absence of Ara h 1 and (**b**) presence of Ara h 1 (control assays), and in the presence of Ara h 1, but in the absence of (**c**) CAb, (**d**) DAb, (**e**) QDs. (**D**) The corresponding DPASV voltammograms. (Experimental conditions: CAb 10 µg·mL^−1^, 250× DAb dilution in Tris BSA 1.0% (m/V)). Other conditions: QDs 5.0 nM + BSA 2.0% (m/V).

After the study/optimization of the previous conditions, the effect of the concentrations of both antibody solutions was studied. Three distinct dilutions were selected for the DAb (500×, 250×, 100×, in Tris BSA 1.0% (m/V)) with a fixed CAb concentration (25 µg·mL^−1^) (Figure 3A). Considering the S/B ratio, a DAb dilution of 250× was chosen to proceed with the development of the immunosensor and several CAb concentrations were tested (50, 25, 10, 5.0, and 2.5 µg·mL^−1^) (Figure 3B), selecting an optimum (highest S/B ratio) concentration of 10 µg·mL^−1^. In fact, the precision and reproducibility observed for the optimum CAb concentration, as well as the obtained *i*_p_ values, suggest that efficient antibody orientation was achieved onto the sensing surface with less steric hinderance.

Before the assessment of the method’s performance characteristics, one of the main issues that can jeopardize the assay’s results was evaluated: the influence of non-specific interactions of the reagents and possible non-specific adsorptions of the target analyte. This was done by testing the assay: (a) without (blank) and (b) with Ara h 1 (250 ng·mL^−1^) (control assays); without the use of (c) CAb, (d) DAb, and (e) QDs (Figure 3C). As expected, the *i*_p_ values obtained for assays (d) and (e) are similar to the blank assay (a). However, without the use of CAb (c) a slightly higher *i*_p_ value was observed, which might be due to the interaction between DAb and the target analyte since a highly specific biotinylated antibody was employed. The control assays ((a) and (b)) confirmed the efficient performance of the developed immunosensor.

The optimized and selected parameters for the analysis of Ara h 1 are indicated in Table S2. In summary, the best performance was obtained using the following conditions: CAb 10 µg·mL-1, mixture of Ara h 1 and DAb (250× dilution, in Tris-BSA 1.0% (m/V) (30 min), and 5.0-nM QD solution containing 2.0% (m/V) BSA (30 min). This protocol allowed the analysis to be carried out in a fast and user-friendly way (total assay time of 1 h with less than 15 min of hands-on time), using a simple transducer without the need of additional complex surface modifications.

### 3.2. Method Performance

**Figure 4:**
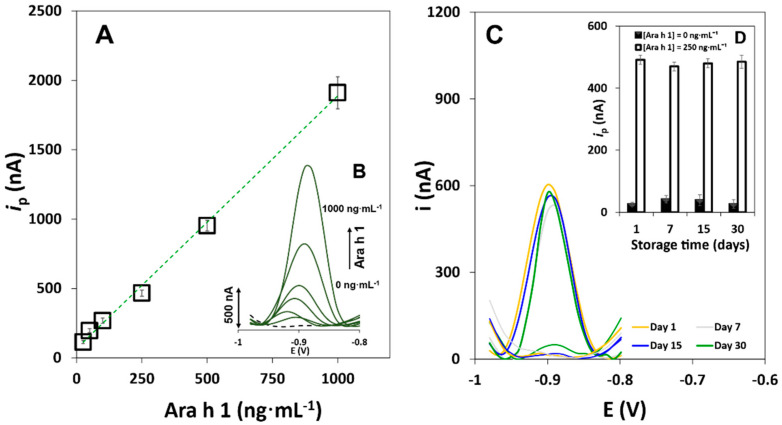
Analysis of Ara h 1. (**A**) Calibration straight; (**B**) Representative DPASV voltammograms ([Ara h 1] (ng·mL^−1^): 0, 25, 50, 100, 250, 500, and 1000). (error bars correspond to the standard deviation of 3 replicates). (**C**) Examples of DPASV voltammograms obtained in the stability study; (**D**) obtained *i*_p_ values in the stability study (day 1 (control), 7, 15 and 30). Experimental conditions: CAb 10 µg·mL^−1^, Ara h 1 (0 and 250 ng·mL^−1^), DAb (250× dilution in Tris-BSA 1.0% (m/V)), QD-strep 5.0 nM + BSA 2.0% (m/V).

A linear range between *i*_p_ and Ara h 1 concentration was established between 25 and 1000 ng·mL^−1^ (*i*_p_ = (1.82 ± 0.05) [Ara h 1] + (66 ± 22), r = 0.998, n = 6, sensitivity = 23.0 nA·mL·ng^−1^·cm^−2^). The calibration straight and representative voltammograms are shown in Figure 4A,B. Additional figures of merit are summarized in Table S3. The values obtained for the LOD (3.5 ng·mL^−1^) and LOQ (12 ng·mL^−1^) were calculated through the equations 3 *s/m* and 10 *s/m*, respectively (where *s* is the standard deviation of 4 consecutively measured blank signals and *m* is the slope of the calibration straight). The precision of the method was acceptable since V_x0_ ≈ 6%.

The stability of the immunosensor was also studied. Distinct SPCEs were modified with CAb according to the optimized protocol. In short, the bioreceptor was immobilized onto bare electrodes and stored at 4 °C in a humidified chamber. The *i*_p_ values were measured during several weeks (electrochemical responses obtained after 7, 15 and 30 days and compared with day 1 (control)), in the presence (S, signal; 250 ng·mL^−1^ Ara h 1) and absence (B, blank; 0 ng·mL^−1^ Ara h 1) of the target analyte. The sensor’s response was stable for at least 30 days, since no significant variations of the *i*_p_ values were observed during this time, maintaining 98.7% of the initial signal (Figure 4C), demonstrating that the CAb affinity to Ara h 1 is not affected over the tested time. The storage conditions such as the temperature and the moist atmosphere contribute to maximize the sensing phase’s long-term stability.

The precision of the results was evaluated using 0 and 250 ng·mL^−1^ Ara h 1 solutions. Intermediate precision and reproducibility were assessed by inter-electrodic measurements (using individual SPCEs) and inter-day assays (n = 3). The corresponding coefficients of variation (CV) were 3.0% and 3.8%, demonstrating that the immunosensor provides precise results.

### 3.3. Ara h 1 Determination in Food Samples

Although certified reference materials are ideal for method validation, because of their absence for Ara h 1 recovery studies were performed to assess the accuracy of the sensor’s results in food sample analysis. A peanut-free product was selected for this purpose and 1.0-g aliquots were prepared and analysed in the absence and presence of Ara h 1 (spiked) (100, 250, 500, and 1000 ng·mL^−1^). Recovery values varied between 102% and 112% (Table S4), demonstrating that the immunosensor provided accurate results. The obtained CV values confirmed the precision of the sensor in the analysis of food products.

Raw peanuts of unknown variety were randomly selected to verify the sensor’s ability to detect Ara h 1. Distinct dilutions were tested and the allergen’s content was quantified (20.4 mg·g^−1^), which is in accordance with the one reported in another study [41].

Additional experiments were performed to assess the selectivity and to evaluate possible food matrix effects. For this purpose, distinct non-target allergens/proteins were used: Ara h 2 (5.0 ng·mL^−1^), Ara h 6 (2.0 ng·mL^−1^), albumin (55 mg·mL^−1^), and ovalbumin (OVA) (100 mg·mL^−1^). The selected concentrations were based on a previous study [42]. and according to the criteria indicated by the standard allergen (Ara h 2 and Ara h 6) supplier (Indoor Biotecnologies, Cardiff, UK). Figure S1 presents the immunosensor’s response in interference studies (light grey bars), that were performed by adding the proteins to Ara h 1 solutions (250 ng·mL^−1^); and in selectivity studies, that were performed in the absence of Ara h 1 (dark grey bars). No significant differences were observed between the controls and the other assays, proving the absence of interferences from the studied proteins and the selectivity of the sensor towards Ara h 1.

**Figure 5:**
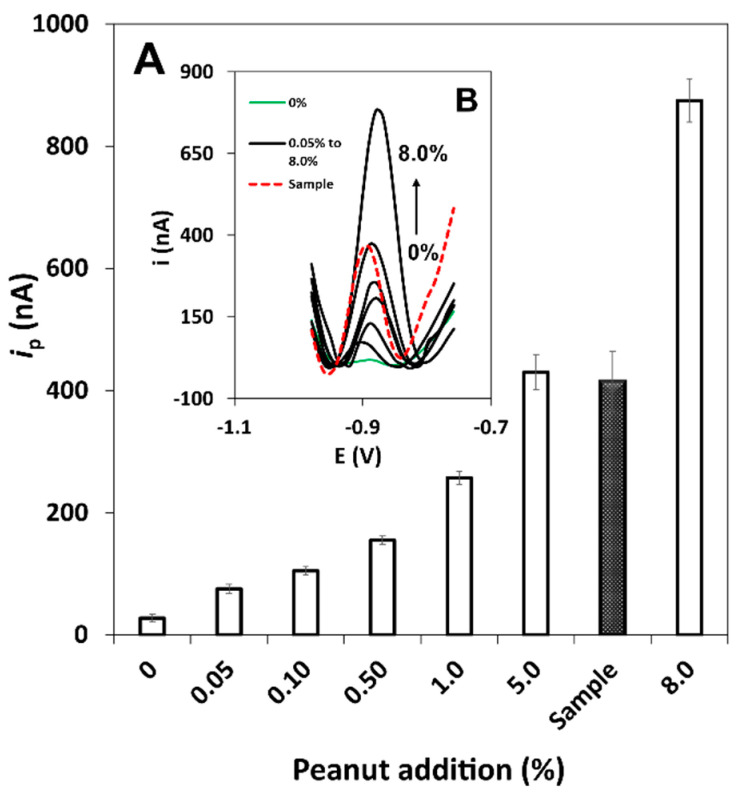
(**A**) Results of the analysis of Ara h 1 in extracts of unspiked cookie (gluten- and allergen-free product) and spiked with increasing percentages of raw peanut (0.05, 0.10, 0.50, 1.0, 5.0 and 8.0% (m/m)), and comparison with a peanut-containing cereal bar (5.5% (m/m)). (**B**) Representative DPASV voltammograms.

Besides this, since casein is an allergenic bovine milk protein, which is often an ingredient in several foods, milk-containing cookies were spiked with increasing amounts of raw peanut (0.05, 0.10, 0.50, 1.0, 5.0 and 8.0% (m/m) and analysed. A protein cereal bar containing a known amount of peanut (5.5%) was also tested. The results obtained is these analyses are shown in Figure 5A, and representative DPASV are displayed in Figure 5B. As can be seen, the sensor clearly allows the detection of at least 0.05% (m/m) of peanut, providing confidence in tracking Ara h 1 in milk-containing food products. The *i*_p_ values obtained for the 5%-spike and the protein cereal bar were in agreement, demonstrating the adequacy of the developed method. The obtained results also allow to confirm that the presence of casein in the analysed product does not interfere since no changes in the signal were observed.

**Figure 6:**
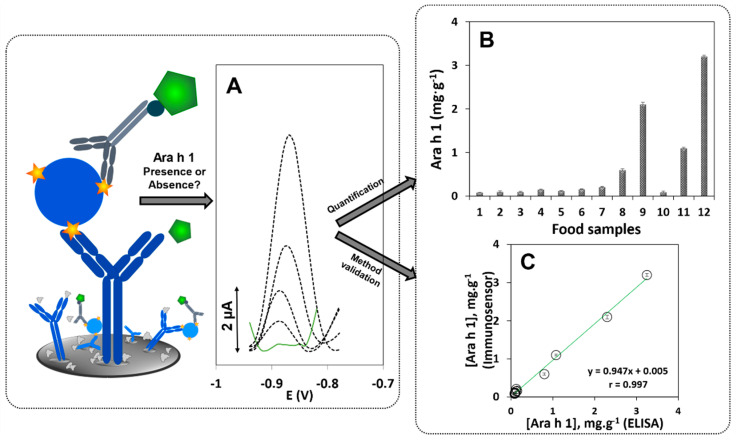
Analysis of food products. (**A**) DPASV voltammograms (dashed line—presence of Ara h 1, solid line—absence of Ara h 1); (**B**) Results of the determination of Ara h 1 in the tested products using the immunosensor; (**C**) Correlation plot between the results of the immunosensor and the ELISA kit. (1) soybean, (2) lupin, (3) almond, (4) sesame, (5) wheat flour, (6) hazelnut, (7) gluten- and allergen-free cookie, (8) cookie that “may contain traces of peanuts”, (9) peanut cookie, (10) cereal bar (No peanut), (11) protein bar, containing 5.5% of peanut, (12) protein bar, containing 12% of peanut.

Tracking food products that may contain traces of peanut or which may be hidden or not mentioned on the label can effectively ensure improved product quality and safety for the consumer. To further test the adequacy of the sensor, a set of commercial food products which clearly state the absence, presence (provided in %) or “may contain traces” of peanut on their labels were tested and analysed. Additionally, legumes and other allergens indicated on the products’ labels were also analysed to verify their possible interference in the analysis. DPASV voltammograms are displayed in Figure 6A and the results are presented in Figure 6B: (1) soybean, (2) lupin, (3) almond, (4) sesame, (5) wheat flour, (6) hazelnut, (7) gluten- and allergen-free cookie, (8) cookie that “may contain traces of peanuts”, (9) peanut cookie (10) cereal bar without peanut, (11) protein bar containing peanut, (12) natural energy cereal bar containing peanut. Ara h 1 was found to be present in the following products: cookie that “may contain traces of peanuts” (0.6 mg·g^−1^), peanut cookie (2.1 mg·g^−1^), protein bar containing peanut (1.1 mg·g^−1^), and the natural energy cereal bar containing peanut (3.2 mg·g^−1^). The low *i*_p_ values obtained in the analysis of the individual ingredients corroborate the selectivity of the proposed immunosensor towards Ara h 1.

A commercial ELISA kit was used to verify the accuracy of the sensor’s results using the previously tested samples. The correlation between the results of both methods indicates the excellent accuracy of the proposed sensor’s results (Figure 6C).

### 3.4. Comparison with Other Electrochemical Immunoassays for the Analysis of Ara h 1

Table 1 summarizes the main characteristics of previously reported electrochemical immunoassays for Ara h 1 analysis [41,43,44,45].

Distinct carbon- and gold-based nanomaterials, magnetic beads and self-assembled monolayers were employed in the sensing surface construction to improve the allergen detection. In the label-free assays the analysis times are shorter than in sandwich-type assays; however, the sensing surface construction requires laborious procedures and a complex sensor platform preparation.

To date no electrochemical immunosensors were reported for the analysis of Ara h 1 using a simplified sandwich-type assay on bare screen-printed electrodes, without a time-consuming sensing surface structuration and/or biofunctionalization with nano- or micromaterials, and using quantum dots as electroactive detection labels. The 1-h assay time (hands on time < 15 min) surpasses the reported sandwich assays (3 h50 min and 2 h assay times) [41,43] and the commercially available kits (ELISA, 2 h assay time).

## 4. Conclusions

Current legislation requires that the presence, even at trace amounts, of peanuts in commercial foods must be declared on the product label. The electrochemical immunosensor developed in this work is useful for the quantification of a major peanut allergen (Ara h 1). The simple, user-friendly, and efficient immunoassay provides high selectivity, precise and accurate results, and an LOD of 3.5 ng·mL^−1^ in a 1-h assay (hands on time < 15 min). The proposed immunosensor was successfully applied to the quantification of Ara h 1 in complex food matrices, such as cookies and cereal and protein bars, and the results were validated using a commercial ELISA kit.

## Figures and Tables

**Table 1:** Summary of electrochemical immunoassays for Ara h 1 analysis.

Sensing Surface		Food Sample	Assay Strategy		LOD	Ref
Construction *	Long-Term Stability		Detection Scheme	Assay Time		
Sandwich-type assay. Capture antibody immobilized on bare SPCE by adsorption (~12 h)	30 days	Cookies, cereal and protein bars	QDs used as label. Detection by DPASV	1 h	3.5 ng·mL^−1^	This work
Sandwich-type assay. Capture antibody immobilized by adsorption on SPCE modified with AuNP (~12 h)	n.d.	Cookies, chocolate	AP used as label. Detection by LSV	3 h 50 min	3.8 ng·mL^−1^	[41]
Sandwich-type assay. Capture antibody immobilized on MBs and using SPCE as transducer (~2 h)	25 days	Hazelnuts, peanut based food; saliva	HRP used as label. H_2_O_2_/HQ system with HQ monitored by amperometry	2 h	6.3 ng·mL^−1^	[43]
Label-free assay. Capture antibody covalently immobilized on a silicon wafer functionalized with SWCNT and (>15 h)	n.d.	n.d.	(Label-free) Detection by LSV	30 min	1 ng·mL^−1^	[44]
Label-free assay. Capture antibody immobilized on 11-MUA/AuE (~19 h)	n.d.	n.d.	Fe(CN)_6_^3−/4−^ used as redox probe. Detection by EIS	5 min	0.3 nM	[45]

3-IP—3-indoxyl phosphate; 11-MUA—11-mercaptoundecanoic acid; Ag^+^—silver ions; Cd^2+^—cadmium ions; AP—alkaline phosphatase; AuE—gold electrode; AuNPs—gold nanoparticles; DPV—differential pulse voltammetry; EIS—electrochemical impedance spectroscopy; H_2_O_2_—hydrogen peroxide; HRP—horseradish peroxidase; HQ—hydroquinone; LSV—linear sweep voltammetry; MBs—magnetic beads; SPCE—screen-printed carbon electrode; QDs—Quantum Dots; SWCNT—single-walled carbon nanotubes; n.d.—no data. * Overnight incubation considered as 12-h period.

## Data Availability

Not applicable.

## Supplementary Materials

The following are available online at https://www.mdpi.com/article/10.3390/bios11110426/s1, Table S1. Time optimization of distinct assay strategies based on a combination of steps; Table S2. Studied and selected variables for the analysis of Ara h 1; Table S3. Analytical characteristics of the developed electrochemical immunosensor for the analysis of Ara h 1; Table S4. Results of the recovery studies; Figure S1. Results from the interference (light grey bars, Ara h 1: 250 ng·mL^−1^) and selectivity (dark grey bars) studies using non-target proteins: Ara h 2 (5.0 ng·mL^−1^), Ara h 6 (2.0 ng·mL^−1^), albumin (55 mg·mL^−1^), and OVA (100 mg·mL^−1^).
