# Supplementary material for: Voltammetric Immunosensor to Track a Major Peanut Allergen (Ara h 1) in Food Products Employing Quantum Dot Labels
Source: Biosensors (Basel). 2021 Oct 29;11(11):426. doi: 10.3390/bios11110426 (PMC8615361; doi:10.3390/bios11110426)
Supplement: Supplementary file 1 [file biosensors-11-00426-s001.zip › biosensors-1408431-supplementary.pdf]

biosensors

MDPI

Supplementary material

Voltammetric immunosensor to track a major peanut allergen (Ara h 1) in food products employing quantum dot labels

Maria Freitas, Henri P.A. Nouws * and Cristina Delerue-Matos

REQUIMTE/LAQV, Instituto Superior de Engenharia do Porto, Instituto Politécnico do Porto, Rua Dr. António Bernardino de Almeida 431, 4200-072 Porto, Portugal; maria.freitas@graq.isep.ipp.pt (M.F.); cmm@isep.ipp.pt (C.D.-M.)

* Correspondence: han@isep.ipp.pt

Table S1. Time optimization of distinct assay strategies based on a combination of steps.

Strategy	Assay step				Assay Time (min)
	**Blocker** (min)	**Target** (min)	**DAb** (min)	**QDs** (min)	
**i**	30	60	60	30	180
**ii**	30	60	60		150
**iii**	30	60		30	120
**iv**	30	60			90
**v**	30	30		30	90
**vi**	--	30		30	60

Table S2. Studied and selected variables for the analysis of Ara h 1.

Experimental variable	Tested range	Selected value
[CAb] (μg·mL^{-1})	2.5 – 50	10
[DAb] (Dilution Factor)	100 – 500	250
t (Ara h 1 & DAb) (min)	30 – 60	30
t (QD-strep) (min)	0 – 30	30
C [QD-strep] (nM)	2.5 – 10	5.0
BSA addition to QD-strep (%)	0.5 – 2.0	2.0

Table S3. Analytical characteristics of the developed electrochemical immunosensor for the analysis of Ara h 1.

Figures of merit	Ara h 1
Concentration interval (ng·mL^{-1})	25 - 1000
Correlation coefficient (r)	0.998
Slope (m) (nA/(ng·mL^{-1}))	1.82
Standard deviation of the slope (S_m) (nA/(ng·mL^{-1}))	0.05
Intercept (b) (nA)	66
Standard deviation of the intercept (S_a) (nA)	22
Standard deviation of the linear regression ($S_{y/x}$)	36
Standard deviation of the method (S_{x0}) (ng·mL^{-1})	20
Coefficient of variation of the method (V_{x0}) (%)	6.1
Limit of detection (LOD) (ng·mL^{-1})	3.5
Limit of quantification (LOQ) (ng·mL^{-1})	12

Table S4. Results of the recovery studies.

Added (ng·mL⁻¹)	Found (ng·mL⁻¹)	Recovery (%)	RSD (%)
100	102	102	6.22
250	262	105	6.79
500	558	112	2.86
1000	1091	109	3.46

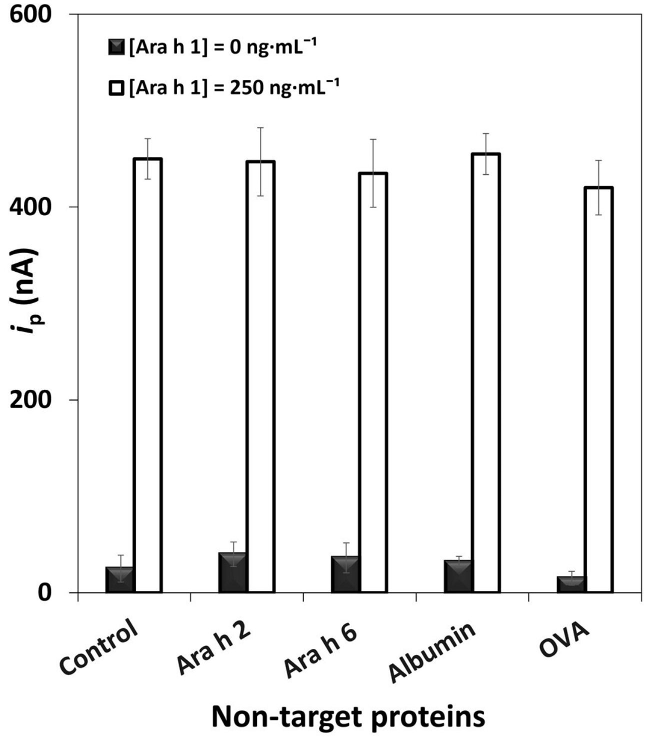

Figure S1. Results from the interference (light grey bars, Ara h 1: 250 ng·mL^{-1}) and selectivity (dark grey bars) studies using non-target proteins: Ara h 2 (5.0 ng·mL^{-1}), Ara h 6 (2.0 ng·mL^{-1}), albumin (55 mg·mL^{-1}), and OVA (100 mg·mL^{-1}).
